# COVID-19 and Cannabis: Playing Saint or Devil?

**DOI:** 10.5152/eurasianjmed.2021.20275

**Published:** 2021-06

**Authors:** Heena Garg, Puneet Khanna

**Affiliations:** Department of Anaesthesiology, Pain Medicine and Critical Care, All India Institute of Medical Sciences, New Delhi, India

Coronavirus disease (COVID-19) pandemic has made everyone haplessly searching for a cure. Various drugs are being put forth to help alleviate the risk of infection and severity. Among these drugs, cannabis is foraying its way up as a potential remedial therapy, leading to a rapid surge in the sale of cannabis. 1 Cannabis is sold legally in many parts of the world for medical purposes. Cannabinoids include delta-9-tetrahydrocannabinol (THC), dronabinol, nabilone, and cannabidiol (CBD). These are used for chemotherapy-induced nausea and vomiting, as an appetite stimulant, for pediatric epilepsy, chronic neuropathic pain, and muscle spasticity.

Cannabinoids have also been advocated to have a possible therapeutic role in viral ailments with a pathogenic host inflammatory. COVID-19 triggers a cytokine storm in the body causing hyper inflammation. Coronavirus uses the angiotensin converting enzyme 2 receptor to enter into the alveolar cells of the lungs. Endocannabinoid system (ECS) is associated with the renin angiotensin system and modulates between the cannabinoid receptor 1 (CB1) and angiotensin II (AT2) levels.^2^ Cannabinoids can potentially target the immune pathways and downregulate the cytokine storm. This is the propelling factor of cannabis use in patients suffering from COVID-19. Costiniuk et al.^3^ and Byrareddy et al.^4^ have proposed the use of CBD as an anti-inflammatory therapy for patients with COVID-19, as cannabinoids regulate the immune system, have anti-inflammatory properties, and have an effect on cardiovascular function and hypertension.

However, this anti-inflammatory activity may not be an advantage when combating viruses because it may mitigate the host immune responses to acute viral and respiratory infections, leading to disease progression and possibly death. Moreover, cannabis smoking is linked with poor respiratory health, immunosuppression, and a higher incidence of stroke in younger individuals. Reece et al.^5^ reported a higher incidence of coronavirus infection with higher use of cannabis and identified it as a risk factor for COVID-19.

There are dichotomous effects of cannabinoids on immunocompetence. Although targeting the ECS for anti-inflammatory role in chronic inflammatory conditions may be beneficial, detrimental effects in the case of acute infections may also be a possibility. Using CBD and THC might reduce the immune response to fight off acute infections, which contrasts against it being touted as a potential anti-inflammatory therapy for coronavirus. Hence, the use of cannabinoids should be avoided and stricter laws should be enforced for regulated sale of these products in this pandemic unless taken for recognized indications.

The ongoing CANDIDATE trial (clinicaltrials.gov Identifier: NCT04467918) – CBD is currently in phase 2 for the use of cannabis plus pharmacological and clinical measures in mild to moderate COVID-19. The trial includes a total of 100 participants infected with coronavirus, 50 in placebo and 50 cases in the CBD group. Patients in CBD group will receive 300mg/day for 14 days. The other ongoing trial is OMNI-Can trial (Outcomes Mandate National Integration with Cannabis) (clinicaltrials.gov Identifier: NCT03944447), which is assessing the efficacy of cannabis for prevention and treatment of COVID-19. The trial is assessing the COVID-19 infection rates in cannabis users compared with those in the general population. It also compares the severity of persistent symptoms and antibodies in cannabis users who become COVID positive in a time frame of 5 years. Further multicentric phase 3 trials need to be conducted before making these drugs available as potential treatment for COVID-19.
